# The use of epigenetic phenomena for the improvement of sheep and cattle

**DOI:** 10.3389/fgene.2014.00247

**Published:** 2014-08-21

**Authors:** Michael E. Goddard, Emma Whitelaw

**Affiliations:** ^1^Department of Food and Agricultural Systems, University of MelbourneParkville, VIC, Australia; ^2^Department of Environment and Primary IndustriesMelbourne, VIC, Australia; ^3^Institute of Molecular Sciences, Latrobe UniversityMelbourne, VIC, Australia

**Keywords:** epigenetics, epimutations, genetic improvement, livestock, sheep, cattle

## Abstract

This review considers the evidence for inheritance across generations of epigenetic marks and how this phenomenon could be exploited in the cattle and sheep industries. Epigenetic marks are chemical changes in the chromosomes that affect the expression of genes and hence the phenotype of the cell and are passed on during mitosis so that the daughter cells have the same chemical changes or epigenetic marks as the parent cell. Although most epigenetic marks are wiped clean in the process of forming a new zygote, some epigenetic marks (epimutations) may be passed on from parent to offspring. The inheritance of epigenetic marks across generations is difficult to prove as there are usually alternative explanations possible. There are few well documented cases, mainly using inbred strains of mice. The epimutations are unstable and revert to wild type after a few generations. Although, there are no known cases in sheep or cattle, it is likely that inherited epimutations occur in these species but it is unlikely that they explain a large part of the inherited or genetic variation. There is limited evidence in mice and rats that an environmental treatment can cause a change in the epigenetic marks of an animal and that this change can be passed on the next generation. If inherited epimutations occur in sheep and cattle, they will already be utilized to some extent by existing genetic improvement programs. It would be possible to modify the statistical models used in the calculation of estimated breeding values to better recognize the variance controlled by epimutations, but it would probably have, at best, a small effect on the rate on genetic (inherited) gain achieved. Although not a genetic improvement, the inheritance of epigenetic marks caused by the environment experienced by the sire offers a new opportunity in sheep and cattle breeding. However, at present we do not know if this occurs or, if it does, what environmental treatment might have a beneficial effect.

## INTRODUCTION

Genetic improvement has been an important method for increasing the profitability of livestock farming for many years. Traditionally it has been based on the use of phenotypic records and pedigrees to estimate the breeding values of individual animals so that those that will produce the most profitable offspring can be chosen as the parents of the next generation. More recently, data on DNA polymorphisms have also been used in the estimation of breeding values as, for instance, in “genomic selection” (Meuwissen et al., 2001). The theory behind these methods assumes that genetic differences between animals are due to differences in DNA sequence inherited in a Mendelian manner. However, there is evidence for epigenetic differences between animals, some of which might be passed on to future generations. The purpose of this review is to explore how across generation inheritance of epigenetic changes could be used for the improvement of sheep and cattle.

The field of epigenetics is large and controversial. Klose and Bird (2006) provide a review of DNA methylation but Ptashne (2013) gives a somewhat different opinion. In this paragraph we very briefly summarize aspects relevant to our topic. During the normal development of an animal, chemical changes occur in the chromosomes that do not change the sequence of nucleotides. These changes include methylation of cytosine bases in the DNA and changes to the histone proteins such as acetylation, methylation and ubiquitination. These changes are called epigenetic marks. They are associated with changes in the expression of genes, that is the transcription of DNA into mRNA. While the order of events is in many cases still unclear, epigenetic changes are associated with turning off a gene in certain tissues where its expression is not required. This is called gene silencing. During differentiation, cells become “committed” to a certain lineage. The inheritance of epigenetic marks through mitosis is thought to be the mechanism by which this commitment occurs. That is, some epigenetic marks are stable across the lifetime of the animal.

Recently it has emerged that these epigenetic changes to chromosomes may also mediate environmental effects on the physiology of the animal. That is, the environmental treatment may affect the phenotype by altering epigenetic marks. For example, gestational exposure to certain nutrients or toxins can permanently affect the epigenetic state and expression of some genes in mice (Wolff et al., 1998; Waterland and Jirtle, 2003; Dolinoy et al., 2006; Kaminen-Ahola et al., 2010). In rats, reduced maternal care immediately after birth can alter the epigenetic state and expression of a gene, the glucocorticoid receptor, in the hypothalamus of the offspring, resulting in them becoming “stressed” adults (Weaver et al., 2004). A recent study in mice has found that parental olfactory experience can influence behavior and neural structures in subsequent generations (Dias and Ressler, 2014). It is not yet clear how extensive this phenomenon is. If, as suggested by the studies cited above, studying the epigenetic state of a gene in newborns allows us to better predict adult phenotype, analysis of epigenome (the epigenetic state of all genes in the genome) will be useful. As described here, this is epigenetic marks mediating a physiological response to the environment – it is not usually a case of inheritance across generations, although examples of inheritance may exist.

Usually epigenetic marks are reprogrammed both during the production of the gametes of the parents and during the formation of a zygote and as a result the zygote acquires the totipotency required to produce daughter cells with the ability to differentiate into any cell type (Santos et al., 2002). However, it is possible for an epigenetic mark not to be erased and therefore to be inherited along with the DNA from parent to offspring (Morgan et al., 1999). It is the importance and implications of this phenomenon with which we are mainly concerned in this review. For instance, if environmental factors can alter the epigenetic state of the genome of animals such that their phenotype is changed and such a change can be inherited across generations, then this could be exploited by those breeding domestic animals.

There are a number of other phenomena which are different from epigenetic inheritance but which tend to be categorised with it in a loose title of non-mendelian inheritance. These include imprinting, cytoplasmic inheritance and transmission of RNA in sperm, and will be briefly reviewed.

In this review, we firstly consider the evidence for transgenerational inheritance of epigenetic marks and then the implications of this for sheep and cattle breeding programs.

## EVIDENCE FOR TRANSGENERATIONAL EPIGENETIC INHERITANCE IN MAMMALS

There is good evidence in a small number of cases in mammals and weak evidence in many more. There is currently no evidence that we know of in either sheep or cattle. The best evidence comes from studies in inbred mouse strains. This does not mean that the phenomenon does not occur in outbred animals but its detection would be more difficult because of the genetic noise.

In genes that show imprinting only one copy (either the paternal or maternal allele) is expressed and the other is silenced. These imprinted genes are an example of an epigenetic mark that is not cleared in the formation of the zygote. However, the mark only lasts for one generation because each generation it is set by whether it is transmitted through a sperm or an egg.

Some transgenes in inbred mice show epigenetic inheritance (Hadchouel et al., 1987; Allen et al., 1990; Kearns et al., 2000). That is, in some animals the transgene is not expressed and their offspring tend to also fail to express the transgene. Because the mice are inbred, it is unlikely that the difference is due to a conventional mutation and so an epimutation is inferred.

Perhaps the best evidence for transgenerational epigenetic inheritance of endogenous (natural) alleles comes from studies of coat color in mice. Inbred mice carrying an agouti allele called A^vy^, show dramatic variation in coat color (Wolff et al., 1998). A^vy^ is a mutation caused by the insertion of a retrotransposon upstream of the agouti locus causing continuous expression of the agouti protein which results in yellow coat color. However, some mice that inherit A^vy^ from their mothers show wild type coat color because the A^vy^ allele has been silenced. This silencing is sometimes passed on to the offspring and is associated with methylation of the retrotransposon promoter (Morgan et al., 1999).

Often transgenerational epigenetic inheritance seems to be associated with methylation of transposons and transgenes, suggesting that its purpose is to protect the genome against invading parasites (Daxinger and Whitelaw, 2010, 2012). The eukaryotic genome is scattered with thousands of mobile elements and many of these proliferate through retrotransposition. Retrotransposition involves the production of RNA intermediates followed by reverse transcription (the production of a DNA copy from the RNA) and integration back into the genome at novel sites. Almost half of the mammalian genome is derived from retroelements (Kalish et al., 2014). In the case of cattle, the figure is around 46% (Adelson et al., 2009). Integration back into the genome is hazardous because it can interrupt the expression of genes at the insertion site, resulting in disease (Ostertag and Kazazian, 2001). The genome has evolved epigenetic mechanisms to suppress and silence these retrotransposons and other invading parasites. It may be advantageous for the epigenetic marks that silence retrotransposons to remain during the reprogramming of the rest of the genome in the gametes and the zygote. There is some direct evidence to support this in the mouse (Lane et al., 2003).

Epigenetic states that are inherited meiotically, independent of an underlying DNA sequence change, are unstable or metastable and lost over a number of generations (Stewart et al., 1980). Studies on this in mammals are rare but this gradual loss has been reported in other model organisms (Cavalli and Paro, 1998).

Other metastable epialleles in mice (e.g., CabpIAP and AxinFu) may also be due to variable methylation of IAP transposons (Faulk et al., 2013).

If an environmental event causes an epigenetic change in the animal and if this change is passed on to the next generation, then inheritance of acquired traits becomes possible. In one instance where environment (gestational exposure to methyl donors) has been shown to influence the coat color of mice, two groups went on to study whether or not these changes were heritable across generations. One group concluded that they were (Cropley et al., 2006) and the other concluded that they were not (Waterland et al., 2007).

Sometimes the term epigenetic inheritance is used loosely and this leads to confusion. For instance, the environment in the uterus may have a long lasting effect on the fetus and part or all of this effect may be mediated by epigenetic changes in the chromosomes of the fetus but this is not transgenerational inheritance of epigenetic marks. For instance, people that were *in utero* during the Dutch famine of 1944 have an impaired glucose tolerance (Lumey, 1992). In mice, under-nutrition during pregnancy caused impaired glucose tolerance and the effect lasted to the second generation. However, it could be that under-nutrition as a fetus affects the maternal environment that a female provides to her young when she matures. To show that this is transmitted through the gametes one could use embryo transfer to separate the embryo from the maternal environment of the female that had experienced under-nutrition as a fetus.

## EVIDENCE FOR OTHER NON-MENDELIAN INHERITANCE

### CYTOPLASMIC INHERITANCE

Mitochondria are passed on in the oocyte from mother to offspring. Mitochondria contain DNA and may influence some traits. However, estimates of the variance that is explained by cytoplamic inheritance are usually <1% of phenotypic variance (e.g., Albuquerque et al., 1998).

### IMPRINTING

A small proportion of genes are imprinted such that only the paternal or only the maternal allele is expressed. Over 70 imprinted loci have been found in mice (Morison et al., 2005). Since there are over 20,000 genes, this suggests that imprinting will explain only a small proportion of the variance. However, there are imprinted genes with a large effect, such as callipyge in sheep (Georges et al., 2003; Lewis and Redrup, 2005). Meyer and Tier (2012) estimated the variance due to imprinted genes to be up to 10% of phenotypic variance for weight traits in beef cattle. However, the effect of maternally imprinted genes is difficult to distinguish from maternal environment effects and the effect of paternally imprinted genes almost vanished when a sire x herd interaction was included in the model. These analyses used only pedigree information. Use of genome-wide SNP genotypes and phenotypes on large numbers of animals should increase our power to distinguish between sources of variation which are partially confounded when only pedigree data is available.

### SEX LINKED INHERITANCE

Sex chromosomes are inherited according to Mendel’s rules but, because many genes on the X-chromosome have no homolog on the Y chromosome, the pattern of inheritance looks different to that of autosomal genes. About 3% of genes are on the X chromosome and so one might expect them to explain 3% of genetic variance although we know of no precise estimates in sheep or cattle.

### RNA IN SPERM

This is a far more controversial topic than the others in this section. If epimutations consist of a metastable change to DNA methylation or a change in the histone proteins, then the effect should act in “cis,” i.e., only the chromosome carrying the epimutation should affect the phenotype. However, there is a small amount of evidence for so-called “trans” effects. For instance, if an allele in the sire that is not passed on to an offspring affects the offspring’s phenotype, this is a trans effect. In this case it could also be called a paternal effect analogous to well-known maternal effects. However, although dams can easily influence their offspring in utero and after birth by the environment they provide, it is less easy to see how a sire, who has almost no contact with the offspring, can influence the offspring, other than through the sperm or semen.

There is circumstantial evidence that these trans effects may be due to RNA transcribed from the sire’s DNA and carried in the sperm (for a review of this see Daxinger and Whitelaw, 2012). Sperm cells carry small amounts of RNA of many different classes including mRNA, endogenous small interfering RNA (siRNA) and PIWI interacting RNA (piRNA). In mouse oocytes siRNA is needed for retrotransposon silencing and piRNAs play a role in imprinting. In fact, it may be that RNA causes changes in DNA methylation which are epigenetic effects.

In summary, there are a great variety of mechanisms of inheritance. Some may be important in specific cases but we do not know at present how important they will be as a source of inherited variation in phenotype. The fact that normal Mendelian laws fit the data so often suggests that other mechanisms are exceptions rather than the rule. The only mechanism, other than conventional Mendelian inheritance, that is commonly included in the analysis of sheep and cattle data is the effect of the environment provided by the dam. For instance, the amount of milk that a cow provides to her calf may well affect the calf’s weaning weight. Unfortunately, it is difficult to distinguish between some of these sources of variation. For instance, a maternal environment effect may be difficult to distinguish from an epigenetic modification inherited from the dam.

## USING EPIGENETIC INHERITANCE FOR GENETIC IMPROVEMENT OF SHEEP AND CATTLE

### CONVENTIONAL SELECTION BASED ON PHENOTYPES AND PEDIGREES

In the calculation of Estimated Breeding Values (EBVs), inheritance is described by the numerator relationship matrix (A) which describes the extent to which relatives resemble each other. The existence of epigenetic inheritance, or the other forms of non-Mendelian inheritance described above, means that the A matrix does not exactly describe the genetic similarity between relatives.

If epigenetic changes (epimutations) were stably inherited then the A matrix would correctly describe the similarity between relatives just as it does for mutations in DNA sequence. However, if epimutations were unstable and lost after a few generations then distant relatives would resemble each other less closely than expected from A. (Unstable mutations in DNA sequence would have the same effect). The A matrix could be modified to reflect this but it is doubtful that the effect on EBVs would be great. Generally distant relatives have only a small effect on EBVs. It would cause estimates of genetic progress to be reduced because it would assume that the genetic gains made in one generation are lost over time. In fact if all mutations were unstable, then long term genetic change would not occur. This is not what we observe in practice which suggests that unstable mutations account for a small part of the genetic (inherited) variance.

### SELECTION USING MOLECULAR DATA

Currently the most important use of molecular data in livestock breeding is genotyping polymorphisms in DNA. These can be causal mutations causing genetic abnormalities or beneficial phenotypes, or random SNPs used for genomic prediction of breeding value (Meuwissen et al., 2001).

If an inherited abnormality was caused by an epimutation it would be impossible to find the cause in DNA sequence data. A DNA sequence polymorphism might still be in linkage disequilibrium (LD) with the causal epimutation and be used as a DNA based test. However, the success in finding the apparently causal mutation for most genetic abnormalities suggests that epimutations are only rarely the cause. Nevertheless this possibility should be kept in mind when it proves very difficult to find a mutation.

Epimutations that are stable will be in LD with SNPs in the same way that DNA mutations are in LD with the SNPs. Therefore genomic selection will still work even if part of the variance in due to stable epimutations.

Unstable epimutations are less likely to be in LD with the SNPs and so will not be included in EBVs calculated using SNP genotypes. It is debatable whether this is a good or bad outcome because selection for unstable mutations is of only short term benefit. The best outcome would be to recognize the unstable mutation and treat it accordingly in selection. It is likely that unstable epimutations account for a small proportion of the genetic (inherited) variance because otherwise long term selection response would not occur. Therefore the gains from including unstable epimutations in genomic selection are likely to be small.

### PREDICTION OF FUTURE PHENOTYPES USING EPIGENETIC DATA

The epigenetic status of sites in the genome can be considered as a phenotype, which depends on the conventional DNA sequence, environmental effects, stochastic events in early development and perhaps inherited epimutations. Therefore epigenetic status could be treated as a selection criterion in the same way that blood concentration of insulin-like growth factor 1 (IGF1) can be used as a selection criterion. Selection for low IGF1 concentration was recommended in cattle to improve feed conversion efficiency but subsequent research has shown this was an unreliable method. We know of no evidence that this would increase the accuracy of selection because it has not been attempted. However, experience of indirect selection criteria such as IGF1 has not been rewarding and epigenetic status may be no better especially if it is not cheap to measure. Perhaps the simplest case to test is whether or not DNA methylation status would predict future phenotype. For instance, if it could predict future marbling phenotype of steers it would be useful, but the test would need to be very cheap (e.g., <$5).

### USING OTHER NON-MENDELIAN OR NON-ADDITIVE INHERITANCE IN GENETIC (HERITABLE) IMPROVEMENT

The A matrix could be modified to account for cytoplasmic inheritance, sex linked genes, imprinting or non-additive variance due to dominance and epistasis. These are all well recognized phenomena but none of them are included in routine genetic evaluation systems in Australia or elsewhere to our knowledge. There are perhaps two reasons why these phenomena have been ignored. Firstly, it is difficult to estimate the variance they explain but it appears to be small (Hill et al., 2008). It is difficult to estimate because the effects are confounded with other effects such as maternal environment effects and even unaccounted for additive genetic variation (Kennedy, 1986). Even if non-additive effects exist, some of the variance they generate is additive genetic variance and so described by the A matrix. Secondly, the gains from utilizing these sources of variation are small. For instance, there is no practical way to utilize cytoplasmic effects because they are not passed on by males and it is bulls and rams that transmit genetic (inherited) gains from studs to commercial herds and flocks.

If the variance due to imprinted genes was as large as some estimates suggest, it could be used by selecting separate sire and dam lines. The sire line would be selected for the performance of offspring when the breed was used as the sire in a commercial cross. This would automatically select favorably imprinted genes and also make some use of non-additive variance, for instance, due to dominance. This form of selection (reciprocal recurrent selection) is used in poultry but seldom in sheep and cattle because it implies selection based on a progeny test using crossbred offspring which may be difficult to organize and which lengthens the generation interval. However, selection based on SNP genotypes (genomic selection) could achieve the same end without an increase in generation length provided the genomic prediction equation was trained using crossbred data.

In general, if there is no experiment by which we can distinguish between alternative hypotheses, there is also no practical situation in which it matters which hypothesis is correct. Therefore one might work backward and ask “in what situations would a better knowledge of the importance of all these sources of variation influence the breeding program adopted?” The selection of sire and dam lines described above is one such situation.

### ENVIRONMENTAL EFFECTS PASSED ON TO FUTURE GENERATIONS

The environment experienced by a female can affect her offspring by means other than inherited epigenetic marks. For instance, under-feeding the female may affect the uterine environment that she provides for her young and their future phenotype. Therefore effects on future generations should be considered when cost: benefit calculations are done on treatments applied to ewes and cows. For instance, the “lifetime wool” project showed that there are benefits in the performance of their lambs from better nutrition of pregnant ewes (Hatcher and Johnson, 2005).

If an environmental treatment of a male affected his progeny’s performance that would be more surprising and more valuable because the treatment would be applied to only a small number of males but benefit the whole herd or flock. This might occur as a result of an inherited epigenetic mark or by RNA attached to sperm. At the moment there is no evidence for this in sheep or cattle and limited evidence in mice but the possibility should not be ignored.

## FUTURE RESEARCH

Inherited epigenetic mutations are difficult to study. The greatest success has been in mice where inbred strains can be used. In the absence of inbred strains it is difficult to prove that an inherited phenotype is not due to a conventional mutation. This helps explain the lack of convincing evidence in humans. Even in mice, there is no case where the mechanism is known. In addition, it is often difficult to rule out other hypotheses such as maternal effects. With these limitations in mind, some suggestions for possible research in sheep and cattle are:

1. * What fraction of genetic variance in economic traits is due to epimutations?* This question is difficult to answer. When the cost of whole genome methylation status becomes cheap enough one could compare the ability of SNPs and epimutations to predict the phenotypes of an animal’s relatives. Genomic selection requires a reference population that has been measured for the trait and genotyped for the SNPs to be used to predict breeding value. The reference population could also be typed for DNA methylation status. However, to have sufficient power, thousands of animals must be typed and so the cost per animal must be low. Practical difficulties involve knowing in what tissue at what age and in what physiological status to record methylation.
2. * What fraction of genetic variation is not explained by DNA sequence?* Given the difficulty of answering question 1 above, it would be worthwhile to know what cannot be explained by DNA sequence. This can be done using the method of Yang et al. (2010) using either SNP genotypes or, in the future, genome sequence. In humans, it is common to find that the additive effect of SNPs only explain 1/3–12 of the genetic variance estimated from the correlation between relatives (Lee et al., 2012). In cattle, 32–80% of the additive genetic variance is explained by the SNPs (Haile-Mariam et al., 2013). One possible explanation for this “missing heritability” is that the mutations that cause variation in a trait are rare and hence in incomplete LD with the SNPs that are genotyped (Yang et al., 2010). If this is the correct explanation, there should be no missing heritability when genome sequence data is used instead of SNP genotypes. Variance not explained by DNA sequence might be due to epimutations but other possible explanations exist, such as upward bias in estimates of heritability from relatives (Zuk et al., 2012).
3. * What fraction of variance is explained by paternal effects?* Using SNP genotypes on sires and their offspring, one could calculate the effect of the sire’s genotype independent of the offspring’s genotype on offspring phenotype. This would indicate the importance of trans effects such as RNA transmitted in sperm. The paternal effect would be partially confounded with the effect of paternally imprinted genes but the two effects are separable. This analysis is feasible given current data on phenotypes and SNP genotypes on sires and offspring.
4. * environmental treatments of males affect their offspring?* This is a doable experiment but we do not know what treatment to impose and what trait to measure. More experimental results in model species such as mice may provide useful guidance in the design of experiments in sheep and cattle.
5. * Can future phenotype be predicted from DNA methylation or more broadly epigenomes?* This is not the main focus of this review because no transgenerational inheritance is involved but it is a more feasible experiment than (1). It will be more feasible when the cost of genome wide methylation assays reduce.
6. * Are there situations in which our prediction of phenotype would be dramatically different if inherited epimutations or other non-additive effects were important?* If there are such situations this would indicate the highest priority experiments. For instance, the effect of sires on crossbred offspring compared with their effect on purebred progeny is one example.

## CONCLUSION

Transgenerational epigenetic inheritance is a “hot” topic scientifically because there is evidence for radically new biological phenomena including inheritance of acquired characteristics. It is likely that these phenomena are as relevant to sheep and cattle as to other mammals. However, they are difficult to study, so that even in humans and mice, their importance is uncertain. Even if transgenerational inheritance of epimutations occurs, it is uncertain how this would affect sheep and cattle breeding programs. For instance, traditional selection on EBVs, calculated as at present, might still be optimal. One clear implication would be that searches for causal mutations should include epimutations. However, we need more information on the amount of variation in phenotype caused by epimutations and have suggested some research approaches that might be feasible even in sheep and cattle.

## Conflict of Interest Statement

The authors declare that the research was conducted in the absence of any commercial or financial relationships that could be construed as a potential conflict of interest.
